# Power Systems and eVTOL Optimization with Information Exchange for Green and Safe Urban Air Mobility

**DOI:** 10.3390/s26030888

**Published:** 2026-01-29

**Authors:** Yujie Yuan, Chun Sing Lai, Hao Ran Chi, Hao Wang, Kim Fung Tsang

**Affiliations:** 1 School of Air Traffic Management, Civil Aviation University of China, Tianjin 300300, China; yjyuan@cauc.edu.cn; 2 School of Electrical and Information Engineering, Tianjin University, Tianjin 300072, China; 3 Instituto de Telecomunicações and Universidade de Aveiro, Campus Universitário de Santiago, 3810-193 Aveiro, Portugal; haoran.chi@ieee.org; 4 Department of Electrical Engineering, City University of Hong Kong, Hong Kong SAR, China; hwang272-c@my.cityu.edu.hk; 5 Shenzhen Institutes of Advanced Technology, Chinese Academy of Sciences, Shenzhen 518055, China; kftsang@ieee.org

**Keywords:** eVTOL, power systems, optimization, flight scheduling

## Abstract

Urban Air Mobility, including electric vertical takeoff and landing vehicles (eVTOL), offer a promising solution to alleviate road traffic congestion and enhance transportation efficiency in cities. However, to ensure its sustainability and operational safety, there is a need for the integrated optimization of eVTOLs and power systems which power these vehicles. Sensors play an important role in data acquisition for the model optimization especially for an environment with high uncertainty. Meanwhile, a quantitative assessment of the eVTOL’s safety level is essential for effective and intuitive supervision. This paper addresses the challenge of achieving both green and safe eVTOLs by proposing an integrated optimization framework. The framework minimizes the costs of eVTOLs and power system operation, and maximizes passenger capacity, by considering the energy stored in the eVTOL as a safety measure. IEEE 2668, a global standard that uses IDex to evaluate application maturity, is incorporated to assess the safety level during the optimization process. A case study for three Chinese cities showed that eVTOLs can utilize inexpensive surplus energy.

## 1. Introduction

The rapid advancement of electric vertical takeoff and landing aircrafts (eVTOL) is poised to revolutionize urban air mobility (UAM) by offering efficient, environmentally friendly transportation alternatives for consumers [1,2]. For instance, the UK has unveiled the UK future of “flight action plan”, ambitiously targeting the first eVTOL flight in 2026 with a fund of GBP 125 billion [3]. As cities are preparing for the integration of eVTOLs into their transportation networks, a critical challenge emerges: the need to effectively manage the increased electrical demand on urban power systems [4,5].

Transportation is a major sector to be decarbonized followed by the residential and industrial sectors. Electric vehicles (EVs) are a mature technology, and the management of EV charging has been well investigated, including the development of charging management strategies to address time anxiety [6], different services [7], and power allocation [8]. Although there have been attempts, merging unmanned aerial vehicles and EVs into the transportation [9] and/or the power system (e.g., [7] smart EV charging [5]), under the fact that eVTOL itself is still in the embryonic stage, the integration of eVTOLs to both sectors is still facing unique and unsolved challenges. Table 1 shows that the operational characteristics of EVs and eVTOLs are inherently different. For the maximum efficacy of eVTOLs, it is crucial to implement a well-organized dispatch system according to key characteristics such as safety. Therefore, establishing a scheduling plan centred around safety and demand is fundamental for maximizing the efficacy of eVTOLs.

Existing studies on eVTOL operations have focused on costs and charging strategies which affect journey time. However, it is unrealistic to overlook safety concerns, such as ensuring the safe take-off and landing of multiple eVTOLs, and accounting for time differences caused by the minimum safe spacing between aircrafts. Ignoring these constraints may result in collisions between aircrafts and obstacles such as other aircrafts. Additionally, the impact of eVTOL operations on power systems with renewable integration has not been thoroughly investigated with respect to energy supply considerations. Driven by research challenges, this paper presents the following contributions:

- Developed an integrated eVTOL and power system optimization framework, relying on the realistic modeling of eVTOL operation. For the first time, power flow analysis was investigated under the influence of eVTOL charging with renewable generation. The marginal costs of the power system were investigated, including solar power generation and changes in travel demand.
- Created an optimization model to determine the optimal daily flight schedules considering multiple types of eVTOLs. IDex defined in IEEE 2668, a frontier global standard for Internet of Things (IoT) maturity evaluation, was newly incorporated to measure the safety degree of eVTOL and define the safety constraints in this optimization model. The schedules are prepared based on travel demand changes due to weather conditions. The schedules consider eVTOL safety operation and operational cost minimization.
- For the first time, investigated the impact of eVTOLs in power system operations considering three different weather conditions. Adverse weather conditions impact on eVTOL scheduling delay and reduce power availability. Recommendations on eVTOL and power system operations are provided.

The remainder of the paper is structured as follows: Section 2 overviews the related work. Section 3 presents the two-stage optimization framework on eVTOL operation and power system optimization. Section 4 presents the case study and results with discussions. Section 5 concludes the work with future research directions.

## 2. Related Work

### 2.1. eVTOL Planning and Operation Optimization

Operational studies for eVTOLs are at an early stage of research, where many are referenced from drone operation. Kim et al. [10] proposed an optimization approach for eVTOLs to determine the optimal flight schedule, considering battery duration under varying temperatures. In other work, battery constraints for drones have been considered by M. Arafat et al. [11] in their joint routing and charging strategy model. The flight range has also been considered. In addition, several types of drones and charging methods were included in the joint routing and charging model created by C. Huang et al. [12] and S. Kim [10] according to real-time travel demand. Z. Ghelichi et al. [13] created a time-slot formulation to determine the viable location of charging stations and resolve the scheduling problem with the aim of minimizing the accumulated journey time of individual trips and accumulated annual cost. The challenge of joint route optimization and scheduling based on hybrid real-time demand was investigated by Z. Wang et al. [14] using an adaptive genetic algorithm. The scheduling problem involving spatio-temporally distributed dynamic demand in long term horizon was transformed into an integer linear problem and was resolved through an iterative algorithm created by Wang et al. [15].

High safety and reliability of aircraft are the basic conditions for the normal operation of eVTOLs. Compared to small drones, eVTOLs have a higher requirement for intelligent operation due to maintaining a high safety level when carrying passengers [16]. With regard to costs as of early 2024, EHang’s two-seat EH216-S costs USD 410,000 per fleet [17]. Taking a high range EV as an example, the Tesla Model S USD 66,490 per fleet [18]. The significant cost difference needs the operators to optimally procure the number of eVTOLs to serve passengers.

Guo et al. [19] presented a problem related to the recovery of eVTOL aircrafts (eVTOL-ARP) specifically addressing scenarios like airport shuttles and intercity flights. A unique aspect of the eVTOL-ARP is the flexibility to defer or cancel charging tasks. To enhance recovery flexibility, an optional charging scheme is proposed. The integration of routing and charging tasks differentiates this problem from traditional aircraft recovery issues, making it significantly more complex. This approach aims to deliver efficient and timely recovery solutions for real-world operations. In another work, Wei et al. [20] conducted a survey on eVTOLs, highlighting the crucial role of autonomous navigation for their successful integration and operation in complex urban environments. The growing density of eVTOLs in urban airspace presents unprecedented challenges for air traffic management systems. Innovative approaches are needed to ensure conflict-free and efficient operations.

### 2.2. Energy Management of eVTOL Charging

Like EVs, eVTOLs rely on power systems to be charged with electricity to complete flight journeys. Recently, several researchers attempted to investigate the energy challenges and optimization methods for eVTOL operation considering energy availability. Zou et al. [21] formulated an energy scheduling problem for a prosumer-based urban area, where prosumers serve as drone charging stations for eVTOLs. The objective is to minimize the overall cost of energy supply–demand imbalances. This problem encompasses two key aspects: (1) the relationships between passengers and eVTOLs, and (2) determining an energy balance strategy through power grid energy scheduling for each prosumer. In other related work, Shihab et al. [22] developed an optimal fleet dispatch framework for eVTOLs designed to transport passengers and provide power grid services, either independently or simultaneously. The main objectives of this framework are as follows: (1) maximizing revenue from passenger transportation, (2) maximizing revenue from frequency regulation services provided to the power grid, and (3) minimizing operating and charging costs. Velaz-Acera, Alvarez-Garcia, and Borge-Diez [23] presented a cost–benefit analysis of the bi-directional functionality of eVTOL charging, using a genetic algorithm to determine the optimal number of eVTOL vehicles for profitable vehicle-to-grid services. The study concluded that increasing the penetration of renewable energy sources leads to a reduction of approximately 4.6% in global emissions. Table 2 compares the recent work which considers the factors of UAM and power grid when managing the UAM operation. It is evident that most UAM operation papers lack consideration of the power grid aspect.

## 3. Framework Integrating eTVOLs and Power Systems

**Figure 1:**
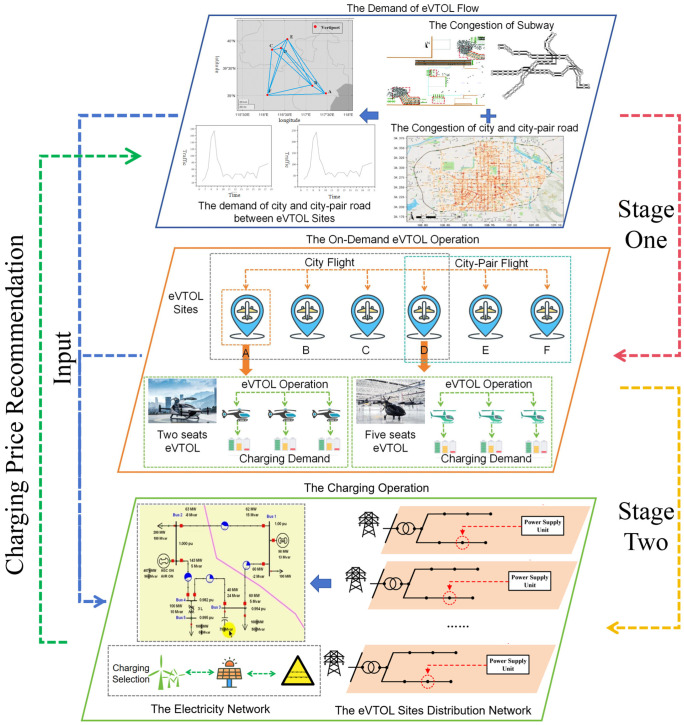
The two-stage optimization framework for eVTOL and power system operation with information exchange.

Figure 1 presents a two-stage optimization framework for the integrated operation of eVTOL and power systems. Stage one performs the optimal scheduling and routing of eVTOLs, considering factors such as flight schedules, passenger demand, and battery charging requirements for eVTOLs. Stage two addresses the power system operation, optimizing the dispatch of electricity generation with power flow analysis and the allocation of charging infrastructures to meet the dynamic demands of eVTOL operations while minimizing operational costs and maintaining power system limits. The output of stage one includes the number of flights that arrived and departed and each vertiport to meet the travel demand at the least operational cost. The output of stage two determines the power system marginal cost which is a recommendation for eVTOL charging price. The details of the optimization framework are presented in the subsequent sections.

### 3.1. eVTOL Operational Optimization Model

Equations (1) and (2) are the objective functions of the maximum served passenger and minimum total cost, respectively. Total cost is composed of purchasing eVTOL, charging and maintenance, where charging cost is the product of charging time *Tc* charging power *Po* and the electricity price *Pe(t)* (Equation (3)). The value of electricity price was set according to the tiered electricity price, which varies based on the energy consumed at different times, *t* (Equation (4)). Purchasing cost is the sum of purchase price of an eVTOL (Equation (5)). Maintenance cost, CM, is calculated based on the travel distance of each eVTOL in Equation (6). As passengers can only wait for *μ*, the demand that could be served by eVTOL *x* is the total demand, acquired through integrating the time–demand function *f_i_* during time *ti-μ* to *ti*, minus the demand that has been already served by other eVTOLs arrives before eVTOL *x* (Equation (7)).

IEEE 2668, a global standard where a maturity index namely IDex is defined, is incorporated to define the safety constraints in this optimization model [24]. IDex provides quantitative results, allowing decision-makers to assess the maturity of an application. In this paper, IDex is used to evaluate the safety level, i.e., a safety index of eVTOLs during operation. By using IDex, users can easily gauge the safety status of eVTOLs, and manufacturers can adjust and enhance their safety performance based on the IDex values. IEEE 2668 is incorporated to define the safety constraints in this optimization model [24]. IDex evaluation quantifies the evaluation results with the designed evaluation criteria, as shown in Table 3.

Table 3 defines the evaluation criteria for Safety IDex (${IDex}_{safety}$) for the remaining electricity level $E_{t}$, passenger number $S_{ij}(t)$. These indicators are denoted as ${IDex}_{E_{t}}$, ${IDex}_{S_{ij}(t)}$. Through Table 3, the IDex safety values can be obtained by filling the obtained $E_{t}$ and $S_{ij}(t)$, in corresponding ranges. For instance, if $E_{t}$ = 76%, ${IDex}_{E_{t}}=4$. As demonstrated in J. Chen et al. [25] research for safety concerns, 30% reserved battery level is required while operating. Hence, ${SDex}_{E_{t}}$ needs to be larger than or equal to level 2 (Equation (8)). The remaining energy *E_t_* in Equation (8) can be obtained through subtracting the energy consumed while flying during (*t-u,t*) from the energy *u* hours ago as Equation (9) illustrates. Equation (10) illustrates that the passenger that eVTOL *i* served should not exceed the upper-bound of its passenger capacity. In other words, ${SDex}_{S_{ij}(t)}$ should be at least level 3. Equation (11) illustrates the takeoff and landing time interval should be beyond the minimum safety interval $T_{s}$. Equation (12) demonstrates each eVTOL can only execute a flight within its maximum range. Alongside that, an eVTOL can charge at will if its remaining state of charge *E_t_* satisfies the electricity required on its next flight but must be charged if its remaining electricity falls below 30% state of charge or cannot meet the requirement of next flight, and the electricity level after charging can’t exceed its battery capacity in Equation (13).

Equation (14) constrains the departure and arrival time lies between the start and final service time. Equation (15) states that the charging time *T^c^* equals 0 when the eVTOL is not charged. *M* is an infinite number. Equation (16) limits the dwelling time of each eVTOL. According to research, UAM can relieve at least 45% ground transportation demand during peak hours [23]; thus, Equation (17) is established to alleviate the ground transportation pressure to the greatest extent. With the evaluation of the IDex for the two indicators, a total IDex can be obtained using Equation (18). ${IDex}_{x,m_{q}}\;$ and $w_{x,m_{q}}$ indicate the IDex values for the evaluated indicator $m_{q}$ (i.e., remaining electricity and passenger number) for the eVTOL x and the weightings, respectively.

A hybrid intelligent optimization method is adopted that integrates the ideas of Particle Swarm Optimization (PSO) and Genetic Algorithms (GA). The algorithm retains the mutation mechanism of the genetic algorithm but does not introduce a crossover operator; instead, random binary variables are used to control whether each chromosome encoding position undergoes mutation. For the mutated encodings, adjustments are made using the update rules of particle swarm optimization, driven jointly by the individual learning factor, the social learning factor, and the inertia weight. In the scheduling optimization stage, the optimal scheduling scheme is obtained by maximizing throughput. On this basis, a cost optimization model is introduced to minimize the total system cost. Finally, based on the Pareto dominance criterion, the optimal eVTOL configuration and scheduling scheme that balances operational efficiency and economic performance is determined. The details with regard to the optimization parameters can be found in the previous work [26]. The formulation is presented as follows:

(1) $$\max\sum_{t=T_{1}}^{T_{2}}\sum_{x=1}^{A_{t}}\sum_{y=1}^{N_{i}}S_{xy}\left(t\right)$$

(2) $$\min C^{c}\left(t\right)+C^{p}+C^{M}$$

where

(3) $$C^{c}\left(t\right)=\sum_{t=T_{1}}^{T_{2}}\sum_{i=1}^{A_{t}}\sum_{j=1}^{N_{i}}T_{ij}^{c}\cdot P^{e}\left(t\right)\cdot Po$$

(4) $$P^{e}\left(t\right)=\left\{\begin{array}{c} R_{1},\\ R_{2},\\ R_{3}, \end{array}\begin{array}{c} \sum_{t=T_{1}}^{T_{2}}\sum_{i=1}^{A_{t}}\sum_{j=1}^{N_{i}}T_{ij}^{c}\cdot Po\leq Thr_{1}\\ Thr_{1}<\sum_{t=T_{1}}^{T_{2}}\sum_{i=1}^{A_{t}}\sum_{j=1}^{N_{i}}T_{ij}^{c}\cdot Po\leq Thr_{2}\\ \sum_{t=T_{1}}^{T_{2}}\sum_{i=1}^{A_{t}}\sum_{j=1}^{N_{i}}T_{ij}^{c}\cdot Po>Thr_{2} \end{array}\right.$$

(5) $$C^{P}=\sum_{y=1}^{B}\sum_{x=1}^{b_{y}}P_{x}^{p}$$

(6) $$C^{M}=\sum_{y=1}^{B}\sum_{x=1}^{b_{y}}Ma\cdot {DT}_{x}$$

(7) $$S_{xy}\left(t\right)=\int_{t_{xy}-\mu }^{t_{xy}}f_{j}dt-\sum_{x=1}^{n}S_{xy}\left(t\right)$$

Subject to

(8) $${SDex}_{E_{t}}\geq 3$$

(9) $$E_{t}=E_{t-\mu }-K_{e}\cdot V_{i}\cdot \mu$$

(10) $${SDex}_{S_{ij}(t)}\geq 3$$

(11) $$t_{i+1j}-t_{ij}\geq T_{s}$$

(12) $$D_{ij}\leq d_{i}$$

(13) $$\max\left\{0.3E_{i}^{0},KD_{ij}\right\}\leq Po\cdot T_{ij}^{C}\cdot r+E_{t}\leq E_{i}^{0}$$

(14) $$T_{1}\leq t_{ij}\leq T_{2}$$

(15) $$0\leq T_{ij}^{C}\leq M\cdot r$$

(16) $$0\leq T_{ij}^{d}\leq 1$$

(17) $$\sum_{t=T_{1}}^{T_{2}}\sum_{x=1}^{A_{t}}\sum_{y=1}^{N_{i}}S_{xy}\geq 0.45\cdot Pd_{yk}$$

(18) $${IDex}_{x,safety}=\sum w_{x,m_{q}}{IDex}_{x,m_{q}}$$

### 3.2. Formulation of the AC-OPF

**Figure 2:**
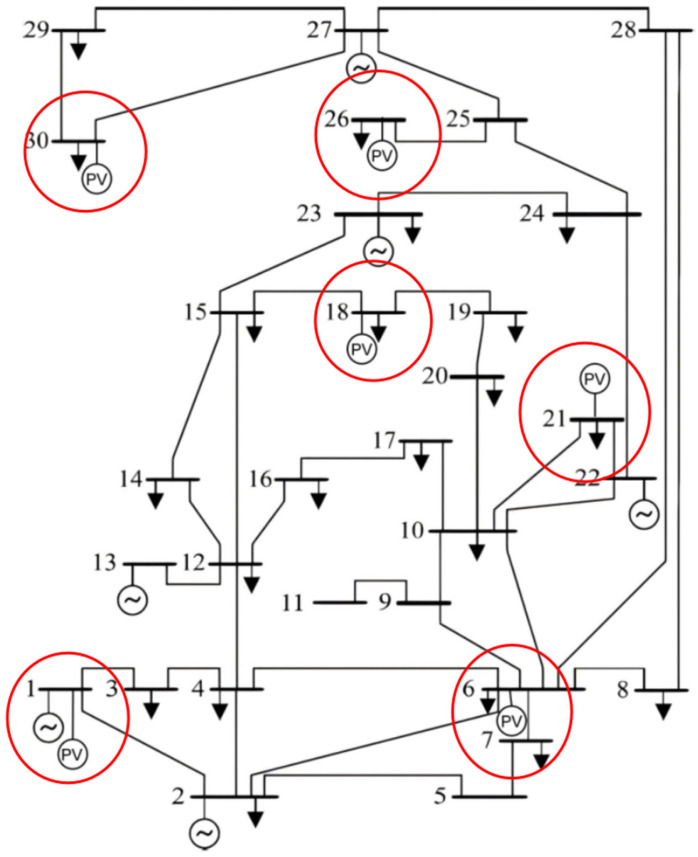
One-line diagram of the IEEE 30-bus system with charging demand and solar generation, as highlighted in red. PV stands for photovoltaic and ~ symbol stands for synchronous generator.

AC Optimal Power Flow (AC-OPF) determines the optimal operating conditions of a power system while satisfying various physical and operational constraints. Figure 2 presents an IEEE 30-bus system [27] which has been modified here. eVTOL vertiports have charging stations installed, as well as solar photovoltaic generators rated at 3 MW for each bus, as highlighted. The solar power is a negative load and offsets the eVTOL charging power.

The objective function aims to minimize the total generation cost, which is expressed in Equations (19) and (20):

(19) $$\underset{p_{g}}{\min}\sum_{g\in G}C_{g}\left(P_{q}\right)$$

(20) $$C_{g}\left(P_{g}\right)=a_{g}p_{g}^{2}+b_{g}p_{g}+c_{g}$$

where *G* is the set of generators. *p_g_* is the real power output of generator *g*. $C_{g}\left(P_{g}\right)$ is the cost function of generator *g*.

The AC-OPF problem includes several constraints, which can be categorized as power balance constraints, generator limits, voltage limits, and line flow limits.

1. Power Balance Constraints

The power balance constraints in Equations (21) and (22) ensure that the generation meets the load demand plus losses:

Real Power Balance:

(21) $$\sum_{g\in G_{n}}P_{g}=d_{n}+\sum_{\left(n,m\right)\in L}f_{nm}^{p}$$

Reactive Power Balance:

(22) $$\sum_{g\in G_{n}}q_{g}=\gamma d_{n}+\sum_{\left(n,m\right)\in L}f_{nm}^{q}$$

2. Generator Limits

Each generator has limits on its real and reactive power outputs, as given in Equations (23) and (24):

Real Power Limits:

(23) $$p_{g}^{min}\leq p_{g}\leq p_{g}^{max},\forall g\in G$$

Reactive Power Limits:
  (24) $$q_{g}^{min}\leq q_{g}\leq q_{g}^{max},\forall g\in G$$
  where $p_{g}^{min}$ and $p_{g}^{max}$ are the minimum and maximum real power outputs of generator g, and $q_{g}^{min}$ and $q_{g}^{max}$ are the minimum and maximum reactive power outputs of generator *g*. $q_{g}$ is the reactive power outputs of generator *g*.

3. Line Flow Limits

The power flow on each transmission line must not exceed its thermal limit as given in Equations (25)–(27):

Apparent Power Flow Limit:

$$\forall \left(n,m\right)\in L:f_{nm}^{p}=v_{n}^{2}g_{nm}-v_{n}v_{m}\left[g_{nm}\cos\left(\theta_{n}-\theta_{m}\right)+b_{nm}\sin\left(\theta_{n}-\theta_{m}\right)\right]$$

(25) $$\forall \left(n,m\right)\in L:f_{nm}^{q}=-v_{n}^{2}\left(b_{nm}+\frac{b_{ch,nm}}{2}\right)$$

(26) $$-v_{n}v_{m}\left[g_{nm}\sin\left(\theta_{n}-\theta_{m}\right)+b_{nm}\cos\left(\theta_{n}-\theta_{m}\right)\right]$$

(27) $$\forall \left(n,m\right)\in L:{\left(f_{nm}^{p}\right)}^{2}+{\left(f_{nm}^{q}\right)}^{2}\leq {\left(s_{nm}^{max}\right)}^{2}$$

where $f_{nm}^{p}$ is the active power flow on the line between buses *n* and *m*, $f_{nm}^{q}$ is the reactive power flow on the line between buses *n* and *m*, and $s_{nm}^{max}$ is the maximum allowable apparent power flow on that line. $g_{nm}$ and $b_{nm}$ are the conductance and susceptance of the line between buses *n* and *m*. $b_{ch,nm}$ is the total charging susceptance on branch between buses *n* and *m*. $v_{n}$ and $\theta_{n}$ are voltage magnitudes and phase angle bus *n*, *L* is the set of lines.

4. Voltage Limits

Voltage magnitudes and phase angle at each bus must be within specified limits, as defined in Equations (28) and (29):

(28) $$v_{n}^{min}\leq v_{n}\leq v_{n}^{max},\forall n\in N$$

(29) $$\theta_{n}^{min}\leq \theta_{n}\leq \theta_{n}^{max},\forall n\in N$$

where $v_{n}^{min}$ and $v_{n}^{max}$ are the minimum and maximum voltage magnitudes at bus *n*, and $\theta_{n}^{min}$ and $\theta_{n}^{max}$ are the minimum and maximum voltage phase angle at bus *n*.

Equation (30) calculates the charging demand power for eVTOLs.

(30) $$P_{t,k}^{Char}=N_{t,k}^{eVTOL}\cdot P_{t,k}^{{eVTOL}_{rated}},\forall t\in T,\forall k\in K$$

$P_{t,k}^{Char}$ is the charging power for eVTOL. *t*/*T* is the index/set of time slots and *k/K* is the index/set of bus system nodes with eVTOL chargers.

## 4. Case Studies

**Figure 3:**
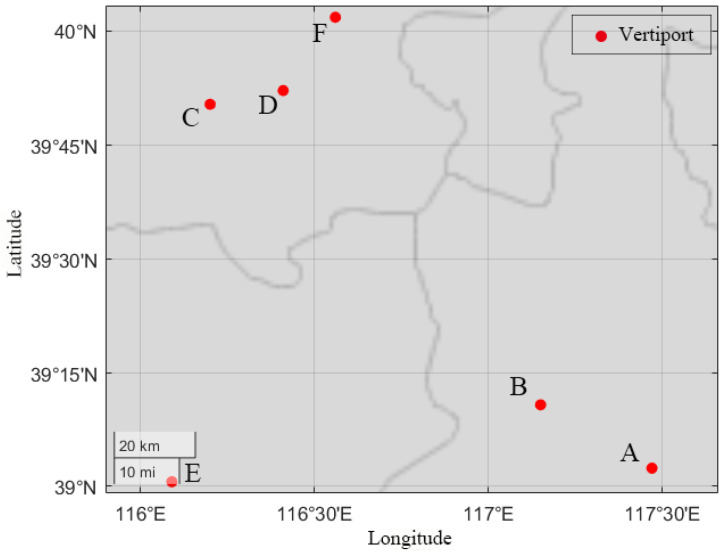
Geographical vertiport location in Beijing (C/D/F)–Tianjin(A/B)–Xiong’an Hebei (E).

This research focused on the Beijing–Tianjin–Xiong’an (Hebei) region, which was selected as a case study due to the high demand resulting from significant ground transportation congestion [18,19]. Vertiport locations were selected based on the demand in each city, with three vertiports in Beijing, two in Tianjin, and one in Xiong’an (Hebei), as depicted in Figure 3. To address safety concerns for citizens, and protect government and military facilities, appropriate detouring measures should be implemented during cruising. According to Civil Aviation Administration of China (CAAC) regulations, the distance between the flight route and the sensitive areas should exceed 10 km [28]. Considering the distance between the busbars for the power system, vertiports A, B, C, D, E, and F correspond to busbars 6, 21, 30, 18, 1, and 26, respectively, in Figure 2. Two eVTOL models are considered in this work, and the specifications are shown in Table 4.

Previous research demonstrated that around 45% of commuters could benefit from urban air mobility on a travel time basis [29]. Case 1 presents the optimization results to meet the 45% travel demand, with Case 2 to be 50% travel demand as

- Case 1: 107 Xpeng X2 eVTOLs and 96 Geely AE200 eVTOLs, with a total 694 passengers’ capacity.
- Case 2: 153 Xpeng X2 eVTOLs and 93 Geely AE200 eVTOLs, with a total 771 passengers’ capacity.

### 4.1. eVTOL Operational Optimization Model Results

**Figure 4:**
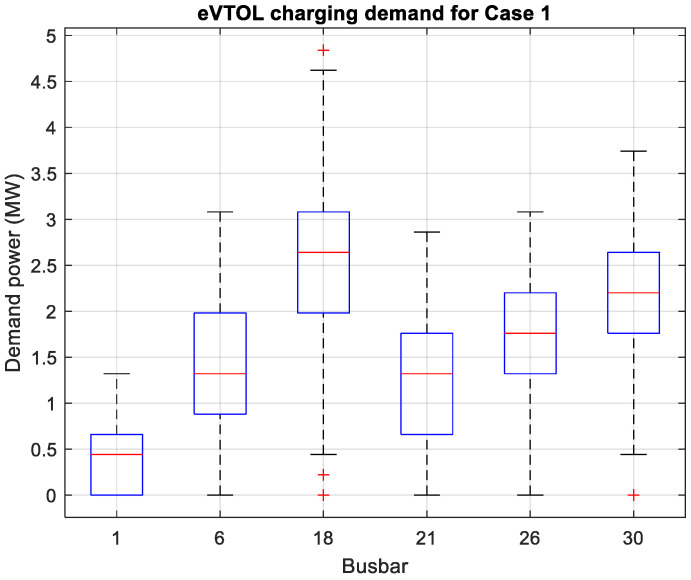
Case 1: eVTOL charging demand with 45% travel demand.

**Figure 5:**
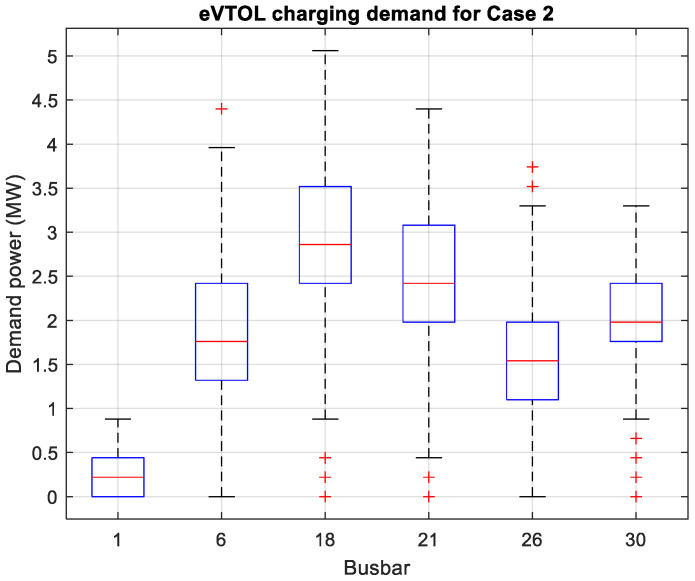
Case 2: eVTOL charging demand with 50% travel demand.

**Figure 6:**
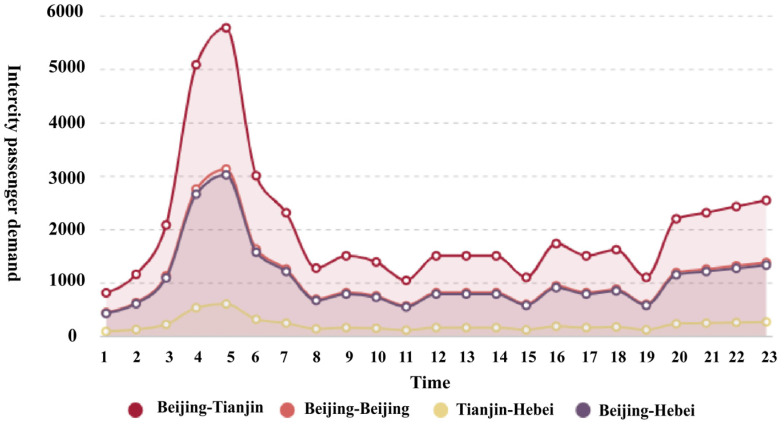
Intercity passenger demand.

Figure 4 and Figure 5 depict the eVTOL charging demand throughout the day for the six buses in two case studies. The boxplots show similar trends with the charging demand the highest for bus 18 (vertiport D) and lowest for bus 1 (vertiport E). This is due to Beijing and Tianjin being popular cities, and Beijing is the most populous city of the three. It should be noted that the boxplots do not reflect the charging time; hence, the idea is to determine the general charging demand trend which can be affected by commute time (e.g., rush hour). With the 5% increase in travel demand, it is shown that busbar 21 (vertiport B) has the biggest increase in charging demand which signifies that more passengers are travelling from Tianjin to other cities. Figure 6 presents the statistics of the intercity passenger demand which are the input data for the power flow model. As shown, the power flow or demand results are highly positive correlated with the intercity passenger demand. As the charging demand from stage one in the framework is realized, the power flow analysis can be conducted in stage two.

### 4.2. IEEE 2668 Compliant Safety Constraints

The optimization process has established IEEE 2668 compliant constraints that these indicators must adhere to. This section presents the changes in IDex for eVTOLs during optimization to verify whether the constraints for remaining electricity and passenger capacity are met. The remaining electricity refers to the battery level of an eVTOL during operation, which, as mentioned earlier, must exceed 30% to ensure safety. Additionally, the passenger count must meet IDex requirements to avoid risks such as overloading.

Additionally, the ${IDex}_{safety}$ for an eVTOL is demonstrated, with weightings equally set for the two indicators. By observing the changing of ${IDex}_{safety}$, the safe degree of the eVTOL can be quantitatively understood.

#### 4.2.1. Single eVTOL

**Figure 7:**
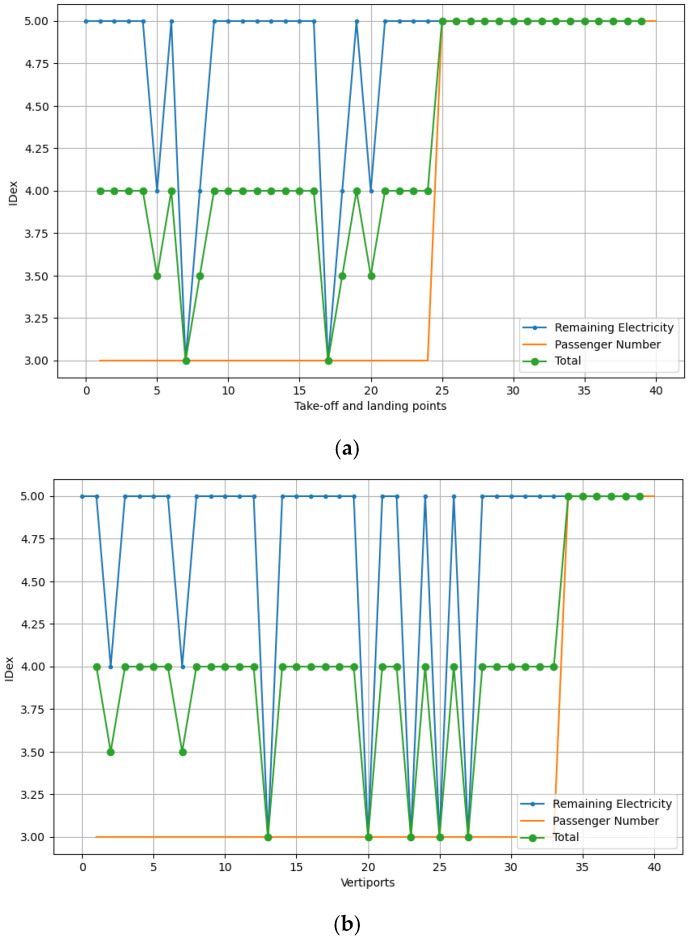
IDex performance for an eVTOL during its operation in 40 vertiports: (**a**) Jili and (**b**) Xpeng.

When an eVTOL moves between different vertiports, there are both battery charging requirements and the need for passenger boarding and disembarking. To ensure safety, the IDex should be greater than 3. From Figure 7a,b, the IDex meets the requirements for remaining battery levels and passenger counts, ranging from 3 to 5. The variation in IDex is due to changes in both remaining battery charge and the number of passengers, as previously mentioned. As a weighted sum of these two factors, the ${IDex}_{safety}$ consistently shows satisfactory values throughout operation. This demonstrates compliance with the IEEE 2668 safety constraints.

#### 4.2.2. Multiple eVTOLs

**Figure 8:**
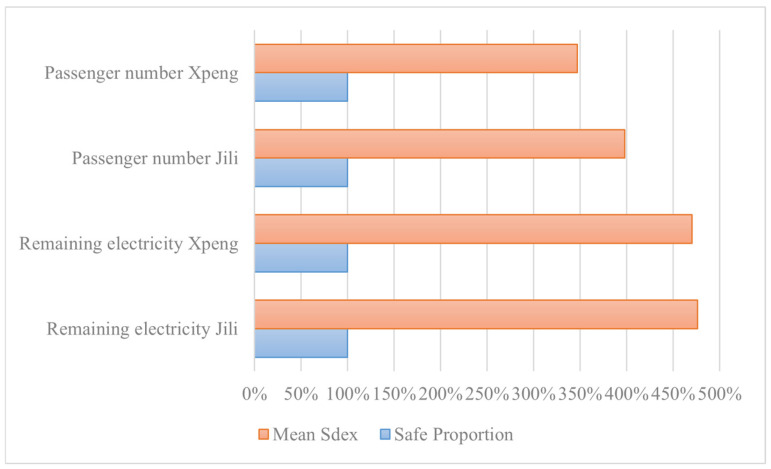
Mean of safety IDex and the proportion of safe eVTOLs.

Figure 8 shows the mean IDex for each indicator and safe proportion. It shows that all IDexs are higher than 3 (the orange column). As the IDex ranges from 0 to 5, it indicates that a satisfactory safety level is achieved. Alongside that, the safety proportion for all indicators achieved 100%, which means there are no outliers during the optimization process. It ensures that the IEEE 2668 compliant safety standards for eVTOLs have been achieved.

### 4.3. Power Flow Analysis

**Figure 9:**
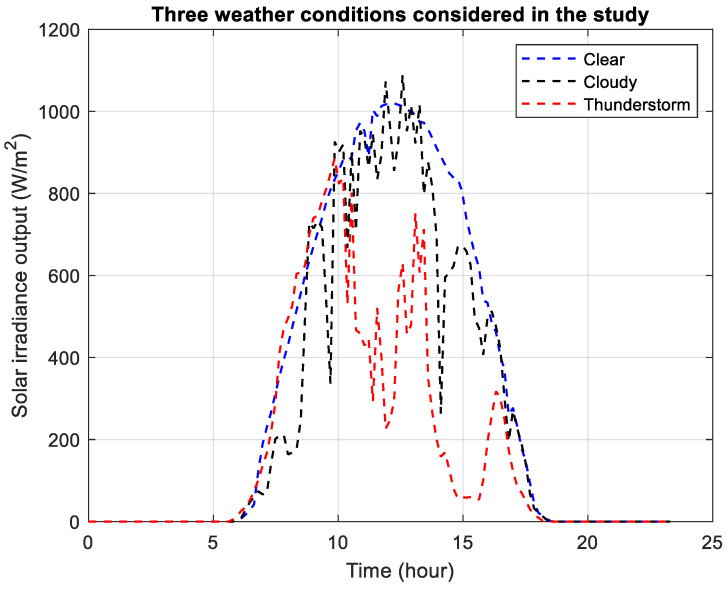
Solar irradiance output scenarios.

Figure 9 presents the solar irradiance output used for the power flow analysis where the three representative days were presented in [30]. The solar irradiance is modeled with the consideration of normal, diffuse, and reflected irradiance. The thunderstorm day shows that the irradiance is reduced during the day with further reduction in the evening.

**Figure 10:**
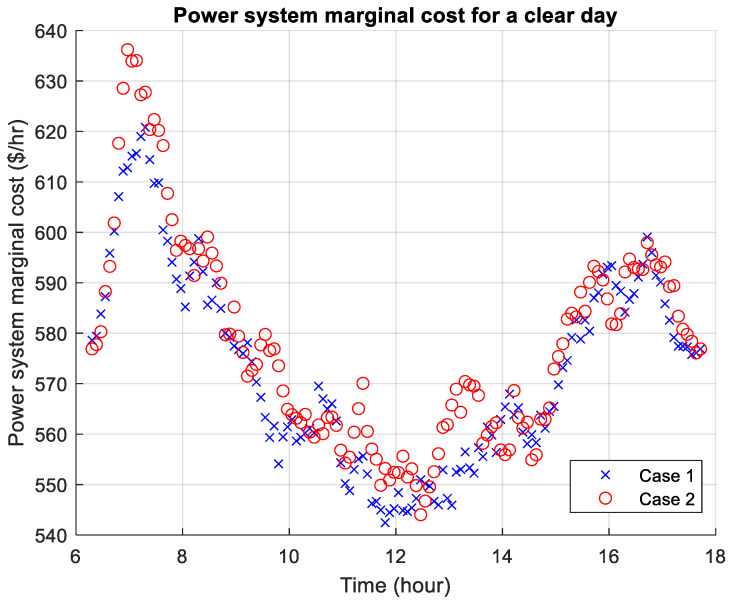
Power system marginal cost for a clear day.

**Figure 11:**
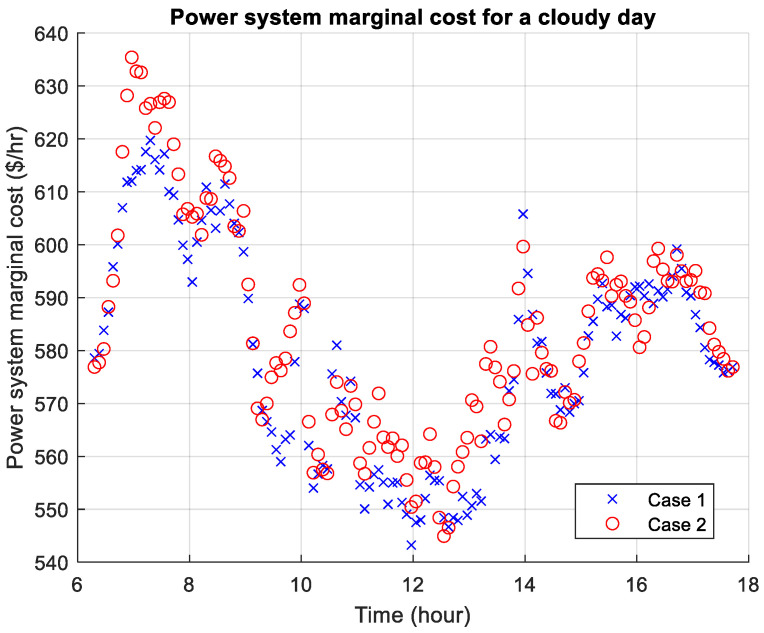
Power system marginal cost for a cloudy day.

**Figure 12:**
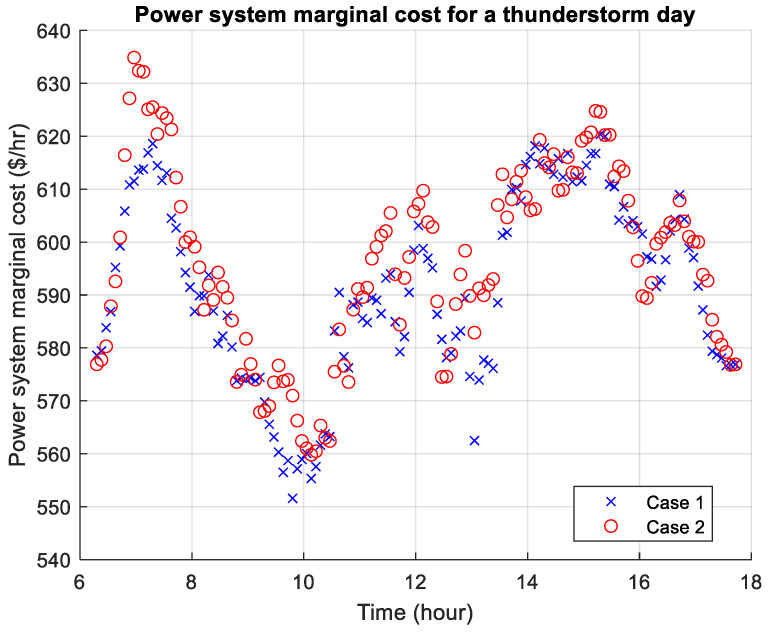
Power system marginal cost for a thunderstorm day.

Figure 10, Figure 11 and Figure 12 present the power system marginal costs according to the three weather scenarios. The high prices during the morning and evening are due to the absence of free solar energy which is abundant during daytime. During a thunderstorm day, the marginal cost can be significantly higher than the other two weather conditions. In comparison, for Cases 1 and 2, Case 2 shows that the marginal price is higher throughout the day, and particularly the morning, which is due to higher travel demand. For the thunderstorm day, there is a higher marginal cost during midday due to poor weather condition and lack of solar energy.

## 5. Conclusions

This work focused on the study of optimizing eVTOL planning and operation with the consideration of power system operation. An innovative two-stage framework considering power system operation is presented. The IEEE 2668 standard was adopted to evaluate and maintain the safety index of eVTOL operations. The results show that the power systems can fulfil the charging demand of eVTOL in a scenario with three Chinese cities, and the marginal generation costs were determined. It is important to realize that the marginal cost can be an indicator for eVTOL ticket prices, similar to conventional aircrafts where fuel prices can affect the ticket price. Considering that power systems’ marginal cost is affected by several factors, including generation surplus and deficit, eVTOL ticket price must consider the marginal electricity cost. Future works could pave way for developing an innovative eVTOL ticket pricing strategy, as well as investigating eVTOLs to grid operation to support power system stability and resilience.

## Figures and Tables

**Table 1:** Comparison of EV and eVTOL technologies, including operational challenges.

	EVs	eVTOLs
**Purpose**	Ground transportation	Aerial mobility
**Design**	Conventional driving	Vertical take-off and landing allow for minimal use of space
**Operators**	Any healthy person with a valid driving license	At present, registered pilot from National Aviation Agencies
**Charging Infrastructure**	Home chargers, public charging stations, and fast-charging networks	Use chargers at vertiports or landing pads, which can be located on rooftops, designated urban areas, or existing helipads
**Travel routes and mode of operation**	On roads and highways with traffic	In sky, with specific routes and air point
**Use cases**	Examples include personal transportation, commercial deliveries, and public transit	Examples include air taxis, medical emergency services, cargo delivery, and regional air mobility
**Battery storage capacity (kWh)**	40–100	100–1000
**Travel range (km)**	Up to 720	About 40
**Payload (kg)**	500–700	100–200
**Charging station power rating**	Up to 350 kW per vehicle	2 MW for 3 eVTOLs
**Highlight Safety features of EVs and eVTOLs**	Battery level and traffic environment	Battery level, traffic environment including taking-off and landing interval

**Table 2:** Comparison of factors considered in UAM and power grids in recent research works.

Reference	Factors Considered in UAM			Factors Considered in Power Grid			Weather Considered?
	Operating Safety	Operating Cost	Passenger or Customer Served	Power Flow	Operating Cost	Renewable Energy Integration	
Kim [10]	No	Yes	Yes	No	No	No	No
Arafat et al. [11]	Yes	No	Yes	No	No	No	No
Huang et al. [12]	No	Yes	No	No	No	No	No
Ghelichi et al. [13]	No	No	Yes	No	No	No	No
Wang et al. [14]	No	Yes	Yes	No	No	No	No
Wang et al. [15]	No	No	Yes	No	No	No	No
Guo et al. [19]	No	Yes	No	No	No	No	Yes
Zou et al. [21]	Yes	Yes	Yes	No	No	Yes	Yes
Velaz-Acera et al. [23]	No	No	No	No	No	No	No
This work	Yes	Yes	Yes	Yes	Yes	Yes	Yes

**Table 3:** Safety IDex for the remaining electricity level and passenger number.

Safety Level	Remaining Electricity Level $E_{t}$	Passenger Number $S_{ij}(t)$
5	$E_{t}\geq$ 80%	$\frac{1}{2}{pc}_{i}{>S}_{ij}(t)$
4	80% $>E_{t}\geq$ 60%	${pc}_{i}{>S}_{ij}(t)\geq \frac{1}{2}{pc}_{i}$
3	60% $>E_{t}\geq$ 30%	$S_{ij}\left(t\right)={pc}_{i}$
2	30% $>E_{t}\geq$ 10%	$2{pc}_{i}{>S}_{ij}(t)>{pc}_{i}$
1	10% $>E_{t}$	$S_{ij}(t)\geq 2{pc}_{i}$

**Table 4:** eVTOL models considered in this work.

	Xpeng X2	Geely AE200
**Maximum speed (km/h)**	130	264
**Number of passengers**	2	5
**Battery capacity (kWh)**	120	250
**Charging time from 0% to 100% state of charge (hour)**	0.6	1.5
**Travel distance (km) and time (hour)**	76 and 0.58	200 and 0.76

## Data Availability

The datasets presented in this article are not readily available because of legal reasons. Requests to access the datasets should be directed to Yujie Yuan.

## List of Symbols

$a_{g},b_{g},c_{g}$	Generator cost constants
$A_{t}$	Total number of takeoff and landing sites in the region (units)
B	Number of eVTOL models
$b_{ch,nm}$	Total charging susceptance on branch between buses *n* and *m*
$b_{x}$	Number of eVTOLs of type *x*
$b_{nm}$	Susceptance of the line between buses *n* and *m*
$C^{c}$	Total charging cost of eVTOLs at the take-off and landing site
*C^M^*	Total maintenance cost
$C^{p}$	Total purchase cost for each type of eVTOL
$C_{g}\left(P_{g}\right)$	Cost function of generator *g*
$d_{n}$	Active power demand at bus *n*
${DT}_{x}$	Travel distance of eVTOL *x*
$D_{ij}$	Distance of the flight between *i* and *j*
$E_{i}^{0}$	Battery capacity upper limit
*E_t−μ_*	Remaining electricity before *μ* the maximum waiting time
*E_t−_*	Remaining electricity level
*f_i_*	Time-demand function
$f_{nm}^{p}$	Active power flow on the line between buses *n* and *m*
$f_{nm}^{q}$	Reactive power flow on the line between buses *n* and *m*
*G*	Generators set
$g_{nm}$	Conductance of the line between buses *n* and *m*
${IDex}_{S_{xy}(t)}$	Safety index for *S_xy_*(*t*)
${IDex}_{E_{t}}$	Safety index for *E_t_*
$KD_{ij}$	Electricity required for the next flight segment
$K_{e}$	Energy consumption per unit distance
*k/K*	Index/set of bus system nodes
*L*	Line set
*m_q_*	Evaluated indicator
*M*	Infinite number
*Ma*	Maintenance cost per km
$q_{g}^{min}$	Minimum reactive power outputs of *g*
*n*	Bus number
$N_{i}$	Number of eVTOLs charging at takeoff and landing site *i* (units)
$Pd_{yk}$	Transportation pressure
*p_g_*	Power output of generator *g*
*p_g_* ^min^	Minimum real power outputs of *g*
*p_g_* ^max^	Maximum real power outputs of *g*
*Pe(t)*	Electricity price
*Po*	Charging power
$P_{t,k}^{Char}$	Charging power for eVTOL
$P_{x}^{p}$	Purchase cost, price per eVTOL of model *x*
$q_{g}$	Reactive power output of generator *g*
$q_{g}^{max}$	Maximum reactive power outputs of *g*
$R_{1}$	Electricity price of the first tier
${SDex}_{E_{t}}$	IDex safety value
*S_xy_*(*t*)	Passenger number
$s_{nm}^{\max}$	Maximum allowable apparent power flow
*t/T*	Index/set of time slots
*T^c^*	Charging time
$Thr_{1}$	Electricity consumption range for the first tier of tiered electricity pricing (kWh)
*t_y_*	Arrival time of eVTOL *y*
$T_{ij}^{C}$	Charging time to complete journey between locations *i* and *j*
*T_S_*	Minimum safety interval
*u*	Hours ago
$V_{i}$	Cruising speed
*v_n_*	Voltage magnitudes of bus *n*
$v_{n}^{max}$	Maximum voltage magnitudes at bus *n*
$v_{n}^{min}$	Minimum voltage magnitudes at bus *n*
$w_{x,m_{q}}$	Weighting
*y*	The *y*-th eVTOL
*μ*	Maximum waiting time
γ	Reactive power coefficient (the reactive-to-active power ratio of bus *n*)
$\theta_{n}^{max}$	Maximum voltage phase angle at bus *n*
*θ* *_n_*	Phase angle of bus *n*
$\theta_{n}^{min}$	Minimum voltage phase angle at bus *n*

## References

[1] Swaminathan N., Reddy S.R.P., RajaShekara K., Haran K.S. (2022). Flying Cars and eVTOLs—Technology Advancements, Powertrain Architectures, and Design. IEEE Trans. Transp. Electrif..

[2] Su J., Huang H., Zhang H., Wang Y., Wang F.-Y. (2024). eVTOL Performance Analysis: A Review From Control Perspectives. IEEE Trans. Intell. Veh..

[3] Transport D.F. (2024). Policy Paper Future of Flight Action Plan. https://www.gov.uk/government/publications/future-of-flight-action-plan.

[4] Wang Z., Younesi A., Liu M.V., Guo G.C., Anderson C.L. (2023). AC Optimal Power Flow in Power Systems With Renewable Energy Integration: A Review of Formulations and Case Studies. IEEE Access.

[5] Li X., Wang Z., Zhang L., Sun F., Cui D., Hecht C., Figgener J., Sauer D.U. (2023). Electric vehicle behavior modeling and applications in vehicle-grid integration: An overview. Energy.

[6] Alsabbagh A., Wu B., Ma C. (2021). Distributed Electric Vehicles Charging Management Considering Time Anxiety and Customer Behaviors. IEEE Trans. Ind. Inform..

[7] Shen J., Wang L., Zhang J. (2021). Integrated Scheduling Strategy for Private Electric Vehicles and Electric Taxis. IEEE Trans. Ind. Inform..

[8] Yan D., Yin H., Li T., Ma C. (2021). A Two-Stage Scheme for Both Power Allocation and EV Charging Coordination in a Grid-Tied PV–Battery Charging Station. IEEE Trans. Ind. Inform..

[9] Huang X., Zhang Y., Qi Y., Huang C., Hossain M.S. (2024). Energy-Efficient UAV Scheduling and Probabilistic Task Offloading for Digital Twin-Empowered Consumer Electronics Industry. IEEE Trans. Consum. Electron..

[10] Kim S.H. (2020). Receding Horizon Scheduling of On-Demand Urban Air Mobility With Heterogeneous Fleet. IEEE Trans. Aerosp. Electron. Syst..

[11] Arafat M.Y., Moh S. (2022). JRCS: Joint Routing and Charging Strategy for Logistics Drones. IEEE Internet Things J..

[12] Huang C.-J., Hu K.-W., Xie B.Z., Cheng H.-W. (2023). A Joint Routing and Charging Management for Drones. Proceedings of the 2023 International Conference on Cyber Management and Engineering (CyMaEn).

[13] Ghelichi Z., Gentili M., Mirchandani P.B. (2021). Logistics for a fleet of drones for medical item delivery: A case study for Louisville, KY. Comput. Oper. Res..

[14] Wang Z., Yu J., Hao W., Xiang J. (2021). Joint Optimization of Running Route and Scheduling for the Mixed Demand Responsive Feeder Transit With Time-Dependent Travel Times. IEEE Trans. Intell. Transp. Syst..

[15] Wang K., Zhang X., Duan L., Tie J. (2021). Multi-UAV Cooperative Trajectory for Servicing Dynamic Demands and Charging Battery. IEEE Trans. Mob. Comput..

[16] Xiang S., Xie A., Ye M., Yan X., Han X., Niu H., Li Q., Huang H. (2024). Autonomous eVTOL: A summary of researches and challenges. Green Energy Intell. Transp..

[17] EHang EHang Unveils US$410,000 Suggested Retail Price for EH216-S Pilotless Passenger-Carrying eVTOL Aircraft in Global Markets Outside China. https://www.ehang.com/news/1049.htm.

[18] Tesla. https://www.tesla.com/.

[19] Guo Z., Hao M., Liu J., Yu B., Jiang Y. (2022). Joint routing and charging optimization for eVTOL aircraft recovery. Aerosp. Sci. Technol..

[20] Wei H., Lou B., Zhang Z., Liang B., Wang F.-Y., Lv C. (2024). Autonomous Navigation for eVTOL: Review and Future Perspectives. IEEE Trans. Intell. Veh..

[21] Zou L., Munir M.S., Hassan S.S., Tun Y.K., Nguyen L.X., Hong C.S. (2024). Imbalance Cost-Aware Energy Scheduling for Prosumers Towards UAM Charging: A Matching and Multi-Agent DRL Approach. IEEE Trans. Veh. Technol..

[22] Shihab S.A.M., Wei P., Shi J., Yu N. (2020). Optimal eVTOL Fleet Dispatch for Urban Air Mobility and Power Grid Services. Proceedings of the AIAA AVIATION 2020 FORUM.

[23] Velaz-Acera N., Arcauz-Durán D., Borge-Diez D. (2023). Life cycle assessment of eVTOL vehicles in island systems. Case Study: Canary Islands. Transp. Res. Procedia.

[24] (2023). Standard for Maturity Index of the Internet of Things—Evaluation, Grading, and Ranking.

[25] Chen J. (2019). Integrated Routing and Charging Scheduling for Autonomous Electric Aerial Vehicle System. Proceedings of the 2019 IEEE/AIAA 38th Digital Avionics Systems Conference (DASC).

[26] Yuan Y., Li J., Zhao X., Wang Y. (2025). Research on eVTOL Scheduling Schemes for Dynamic Demand and Variable Interval. Acta Aeronaut. Astronaut. Sin..

[27] Illinois Center for a Smarter Electric Grid (ICSEG) IEEE 30-Bus System. https://icseg.iti.illinois.edu/ieee-30-bus-system/.

[28] State Council of China (2001). General Flight Rules of the People’s Republic of China (2001 Revision). https://lawinfochina.com/display.aspx?id=12839&lib=law.

[29] Bulusu V., Onat E.B., Sengupta R., Yedavalli P., Macfarlane J. (2021). A Traffic Demand Analysis Method for Urban Air Mobility. IEEE Trans. Intell. Transp. Syst..

[30] Lai C.S., Jia Y., McCulloch M.D., Xu Z. (2017). Daily Clearness Index Profiles Cluster Analysis for Photovoltaic System. IEEE Trans. Ind. Inform..

